# Procyanidin Promotes Translocation of Glucose Transporter 4 in Muscle of Mice through Activation of Insulin and AMPK Signaling Pathways

**DOI:** 10.1371/journal.pone.0161704

**Published:** 2016-09-06

**Authors:** Yoko Yamashita, Liuqing Wang, Fumio Nanba, Chiaki Ito, Toshiya Toda, Hitoshi Ashida

**Affiliations:** 1 Department of Agrobioscience, Graduate School of Agricultural Science, Kobe University, Kobe, Hyogo 657–8501, Japan; 2 Fujicco Co. Ltd, Research Development, Kobe, Hyogo 650–8558, Japan; Tohoku University, JAPAN

## Abstract

Procyanidins are the oligomeric or polymeric forms of epicatechin and catechin. In this study, we isolated and purified dimer to tetramer procyanidins from black soybean seed coat and investigated the anti-hyperglycemic effects by focusing on glucose transporter 4 (GLUT4) translocation and the underlying molecular mechanism in skeletal muscle of mice. The anti-hyperglycemic effects of procyanidins were also compared with those of monomer (−)-epicatechin (EC) and major anthocyanin, cyanidin-3-O-β-glucoside (C3G). To investigate GLUT4 translocation and its related signaling pathways, ICR mice were orally given procyanidins, EC and C3G in water at 10 μg/kg body weight. The mice were sacrificed 60 min after the dose of polyphenols, and soleus muscle was extracted from the hind legs. The results showed that trimeric and tetrameric procyanidins activated both insulin- and AMPK-signaling pathways to induce GLUT4 translocation in muscle of ICR mice. We confirmed that procyanidins suppressed acute hyperglycemia with an oral glucose tolerance test in a dose-dependent manner. Of these beneficial effects, cinnamtannin A2, one of the tetramers, was the most effective. In conclusion, procyanidins, especially cinnamtannin A2, significantly ameliorate postprandial hyperglycemia at least in part by promoting GLUT4 translocation to the plasma membrane by activating both insulin- and AMPK-signaling pathways.

## Introduction

Hyperglycemia and impaired insulin action and/or insulin secretion are associated with macro- and microvascular complications of high morbidity and mortality [1]. Patients with hyperglycemia and/or insulin resistance are also associated with increased risk of cardiovascular disease [2, 3]. Chronic hyperglycemia has become a serious problem in many countries, which is attributed to over-eating and physical inactivity. Owing to widespread changes in dietary intake, the incidence of obesity, hyperglycemia and insulin resistance has been increasing around the world. Therefore, preventing hyperglycemia and improving insulin resistance are important issues for health promotion. It is now generally accepted that bioactive compounds in food can contribute to alleviating chronic diseases including hyperglycemia and insulin resistance.

Procyanidins are the oligomers and polymers of flavan-3-ols consisting of epicatechin and catechin units [4] and are usually found in fruits and other plants. There is increasing evidence that procyanidins possess beneficial health effects including the prevention of hyperglycemia and diabetes mellitus. For example, grape seed procyanidin extract suppressed hyperglycemia in type 2 diabetic rats [5], and cacao and black soybean seed extracts containing rich procyanidins also suppressed hyperglycemia and obesity in high-fat diet-fed mice [6, 7]. However, the underlying molecular mechanisms by which procyanidins suppress hyperglycemia are not yet fully understood.

Glucose transporter 4 (GLUT4) is a major glucose transporter expressing specifically in skeletal and cardiac muscles and adipose tissue and plays a pivotal role in glucose homeostasis by regulating cellular glucose uptake in these tissues. Skeletal muscle accounts for about 75% of insulin-stimulated whole-body glucose uptake [8]. It is reported that insulin-regulated glucose uptake decreases mainly in the skeletal muscle of type 2 diabetes mellitus patients [9]. Thus, muscle is the main tissue for maintaining postprandial glucose homeostasis through the action of GLUT4.

For the uptake of large amounts of glucose into the muscle cells, insulin stimuli and muscle contraction promote translocation of GLUT4 from intracellular storage vesicles to the plasma membrane [10]. Binding of insulin to the insulin receptor (IR) induces phosphorylation of its tyrosine kinase domain, followed by phosphorylation of multiple tyrosine residues on insulin receptor substrate (IRS) molecules. Activated IRS-1 phosphorylates the p85 regulatory subunit of phosphoinositide 3-kinase (PI3K), which phosphorylates phosphoinositide-dependent kinase 1 and downstream Akt and atypical protein kinase C. Finally, the signals transmit to GLUT4 in intracellular storage vesicles for its translocation to the plasma membrane. GLUT4 translocation in skeletal muscle is also stimulated by exercise and muscle contraction via the activation of AMP-activated protein kinase (AMPK) as an insulin-independent signaling pathway [11, 12]. AMPK acts as a cellular energy sensor and regulates metabolic homeostasis. Consequently, there is increased interest in developing AMPK activators as potential therapies for diabetes and obesity [13, 14].

Insulin- and AMPK-signaling pathways are the major regulators of GLUT4 translocation in muscle [15]. Several studies have demonstrated that polyphenols promote translocation of GLUT4 by activating these signaling pathways in peripheral tissues [16, 17]. For example, procyanidin-rich cacao extract [6, 18], resveratrol [19], and anthocyanin [20, 21] were reported to promote GLUT4 translocation through the activation of AMPK. In addition, (−)-epigallocatechin-3-gallate (EGCg) inhibited dexamethasone-induced insulin resistance by activating both PI3K/Akt and AMPK pathways in rat L6 cells [22]. It was also reported that EGCg improves glucose uptake by activating both insulin- and AMPK-signaling pathways in high-glucose-induced insulin-resistant HepG2 cells [23]. In muscle cells, EGCg at 1 nM increased glucose uptake accompanied by GLUT4 translocation depending on PI3K pathway [24]. These results suggest that polyphenols or polyphenol-rich food materials will improve hyperglycemia and insulin resistance through translocation of GLUT4, although the suggested molecular mechanisms and related signaling pathways for the translocation are controversial. In this study, we isolated and purified dimer to tetramer procyanidins from black soybean seeds and investigated GLUT4 translocation and its underlying molecular mechanism in skeletal muscle compared with monomer, (−)-epicatechin (EC) and cyanidin-3-O-β-glucoside (C3G). We further confirmed their anti-hyperglycemic effects by performing an oral glucose tolerance test.

## Materials and Methods

**Fig 1:**
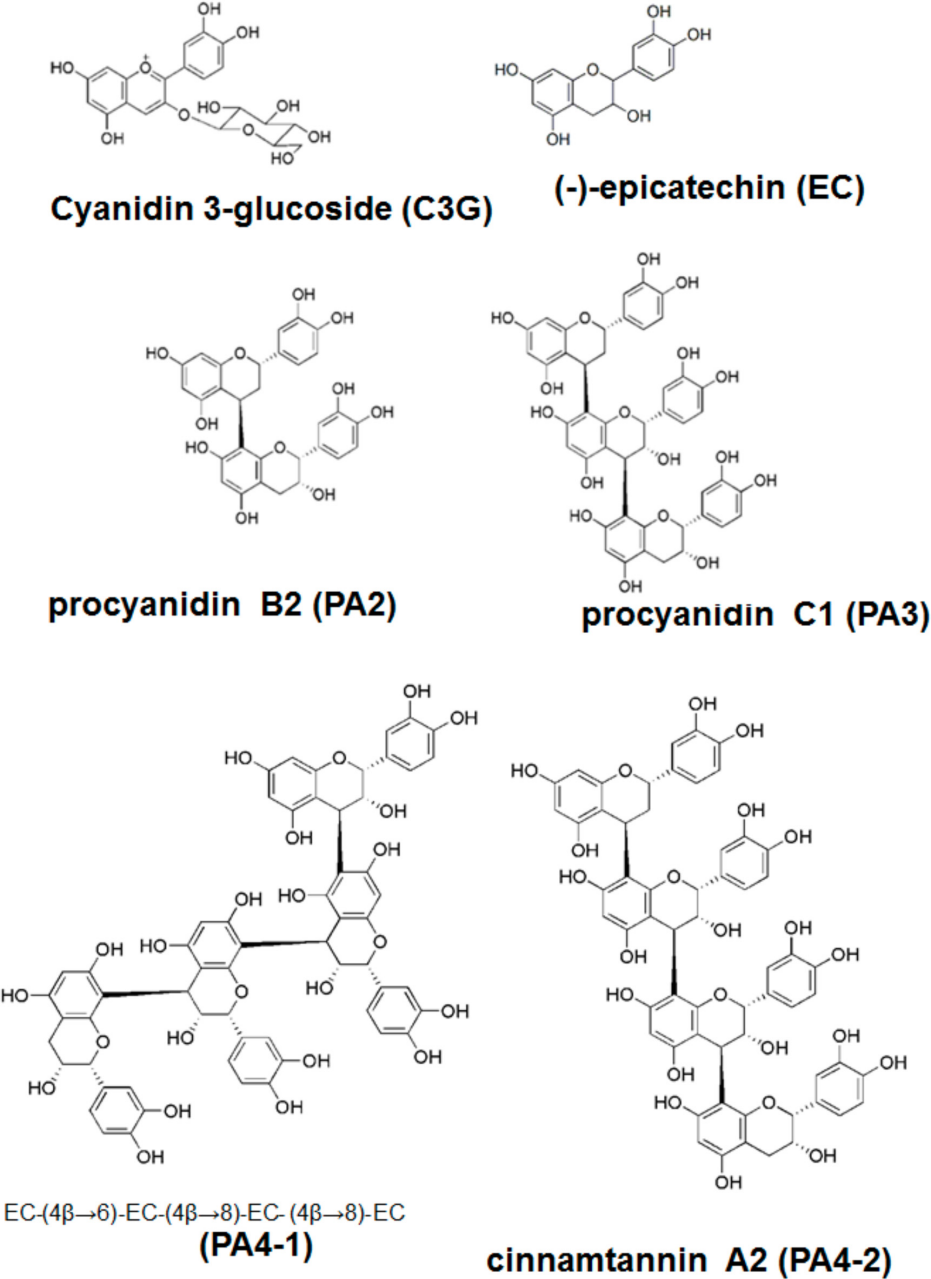
Chemical structures of cyaniding-3-glucoside (C3G), epicatehin (EC) and procyanidins (dimer to tetramer).

*Materials-* (+)-Catechin and EC were purchased from Sigma-Aldrich Co. LLC. (St Louis, MO, USA) and C3G chloride was a product of Fujicco Co., Ltd., (Kobe, Japan). For isolation of procyanidins, commercially available black soybean seed coat extract (BE) (ChoronoCare^®^, Fujicco) was used in this study. BE contains abundant polyphenols including procyanidin oligomers: 6.11% EC, 6.06% procyanidin B2 (PA2), 3.94% procyanidin C1 (PA3), 1.18% cinnamtannin A2 (PA 4–2), 0.76% structural isomer of tetramer EC-(4β–6)-EC-(4β–8)-EC-(4β–8)-EC (PA 4–1) and 5.63% C3G. Percent of total polyphenols and flavan-3-ols concentrations in BE was 68.7% and 40.7%, respectively. Chemical structures of used polyphenols are shown in Fig 1.

Glucose was measured using a commercial kit (Labassay^®^ Glucose Wako kit, Wako Pure Chemical Industries, Ltd., Osaka, Japan). Plasma insulin and active adiponectin levels were measured using corresponding ELISA kit from Shibayagi Co. (Gunma, Japan). Anti-GLUT1 goat polyclonal antibody (#SC-1605), anti-GLUT4 goat polyclonal antibody (#SC-1608), horseradish peroxidase-conjugated anti-goat (#SC-2020), anti-rabbit (#SC-2030) and anti-mouse (#SC-2005) IgG antibodies were obtained from Santa Cruz Biotechnology Inc. (Santa Cruz, CA, USA). Anti-IRS-1 phosphorylation (#2381), anti-AMPKα (#2532), anti-phospho-AMPKα (#2531), anti-PI3K (#4257), anti-phospho-PI3K (#4228) and anti-Akt1 (#9272), anti-phospho-Akt1 Serine 473 (#9271) and Threonine 308 (#9275) were purified rabbit polyclonal antibody and purchased from Cell Signaling Technology Inc. (Danvers, MA, USA). IRS-1 mouse polyclonal antibody (#611395) were product of Becton, Dickinson and Company (Franklin Lakes, NJ, USA). All other reagents used were of the highest grade available from commercial sources.

*Isolation of procyanidins from BE*-The procyanidin oligomers, PA2, PA3, PA4-2 and PA4-1, were isolated from BE. This was done using 6 g of BE applied to a Sephadex LH-20 column (i.d. 10 × 55 cm, GE Healthcare Japan Corp., Tokyo, Japan) pre-equilibrated with 45% methanol and then sequentially washed with 5.2 L of 45%, 55% and 65% methanol, and eluted with 75% and 85% methanol (flow rate 18 ml/min). Three elute fractions were collected and monitored at UV 280 nm: PA2 was eluted with 75% methanol, PA3 with both 75 and 85% methanol and PA4-1 and PA4-2 by 85% methanol. The yield from these fractions was 421, 376 and 268 mg, respectively. The fractions were dried *in vacuo*, and then further purified by a repeating preparative reversed phase HPLC using a Unison US-C18 column (i.d. 20 × 250 mm, 5 μm, Imtakt Corp., Kyoto, Japan) with the mobile phase solvent (A) 0.1% formic acid and (B) acetonitrile. The detection was at UV 280 nm and the flow rate was 8.9 ml/min. For the isolation of PA2 and PA3, elution was carried out using an isocratic mode of 15% B, and for PA4-1 and 2 it was performed using the linear gradient mode of 5 to 20% B in 80 min. Both of the Sephadex LH-20 column chromatography and preparative HPLC were performed using an AKTA purifier 100 system (GE Healthcare Japan Corp.). Finally, 131 mg of PA2, 41 mg of PA3, 7 mg of PA4-1 and 5 mg of PA4-1 were obtained with a purity of ≥95%. The PA2 was identified by direct comparison with the authentic compounds using LC-MS/MS (API2000, AB Sciex, Foster City, CA, USA). The other compounds were identified by comparison with the spectral data from ^1^H NMR and LC-MS/MS in previous reports [25, 26].

*Animal treatment*-All animal experiments were approved by the Kobe University Institutional Animal Care and Use Committee (Permission # 24-04-02) and carried out according to the guidelines for animal experiments at Kobe University Animal Experimentation Regulation. Male ICR mice (4 weeks old) were obtained from Japan SLC, Inc. (Shizuoka, Japan) and maintained at 23 ± 2°C with a 12:12-h light/dark cycle (lights on at 09:00). Five or six mice were kept in each cage. The mice were acclimatized for 7 days with free access to a standard mouse diet (3.850 kcal/g) consisting of 76% carbohydrate, 15% protein and 9% fat (Research Diets, Tokyo, Japan) and distilled water. These mice were subjected to an oral glucose tolerance test (OGTT) and for the detection of GLUT4 translocation and its related signal pathways.

The OGTT test consisted of procyanidins, EC, C3G at 1 ng to 1 mg/kg body weight and water alone (5 mL/kg body weight) as a vehicle control and was orally administered to the ICR mice (six mice in each group) after 18 h fasting. After 60 min, the mice were orally given 1 g/kg body weight of glucose. Blood was collected from the tail vein in heparinized tubes at 0 (before administration), 15, 30, 60 and 120 min after the glucose load and centrifuged at 9,600 × g for 10 min at 4°C and the plasma collected [18]. Plasma was subjected to the measurement of the glucose level using the commercial kit.

For the measurement of GLUT4 translocation and its related signal pathways, another 42 ICR mice were divided at random into seven groups of six each. They were given an oral dose of procyanidins, EC, and C3G in water at 10 μg/kg body weight after 18 h fasting. Mice in the control group received water alone (5 mL/kg body weight). The mice were sacrificed 60 min after the administration of polyphenols under anesthesia using sodium pentobarbital, and euthanized by exsanguination from cardiac puncture. Plasma was collected and the glucose, insulin and adiponectin levels were measured using the corresponding commercial kit. The soleus muscle was collected from the hind legs and its plasma membrane fraction and tissue lysate were prepared and subjected to western blotting [27]. The small intestine was also collected and subjected to the measurement of α-glucosidase activity.

*Immunoblotting*-Proteins in the plasma membrane fraction and tissue lysate were separated by SDS-polyacrylamide gels and transferred to the polyvinylidene difluoride membranes. After blocking with Blocking One^®^ solution (Nacalai Tesque, Kyoto, Japan), the membranes were incubated with the specified primary antibodies overnight at 4°C, followed by the corresponding horseradish peroxidase-conjugated secondary antibody for 1 h at room temperature. The proteins were visualized using ImmunoStar^®^ LD (Wako Pure Chemical Industries, Ltd.) and detected with Light-Capture II (ATTO Corp., Tokyo, Japan).

*Measurement of α-glucosidase activity in the small intestine*-α-Glucosidase activity was measured in the small intestine of mice as described previously [18]. The intestinal mucosa was removed and homogenized with three volumes of 1.15% (w/v) KCl solution on ice. The homogenate was centrifuged at 1,000 × g for 10 min at 4°C, and the resultant supernatant was collected and used to measure maltase and sucrase-isomaltase activities [18].

*Measurement of flavan-3-ols in plasma of mice given BE*- For analysis of plasma concentration of procyanidins, six ICR mice were divided into two groups of three each. They were either given an oral dose of BE in water at 1 g/kg body weight or water alone (5 mL/kg body weight) as a vehicle control after 18 h fasting. Another eighteen ICR mice were divided into 6 groups of three each and used for measurement of flavan-3-ols after administration of EC, PA2, PA3, PA4-1 or PA4-2 in water at 10μg/kg body weight or water alone (5 mL/kg body weight). The mice were sacrificed 60 min after the dose of BE or each flavan-3-ol and blood was collected from a cardiac puncture. An aliquot of 300 μl of plasma was incubated with 250 μl of β-glucronidase in 0.1 M acetic acid buffer (pH 5.0) and 50 μl of 20% (w/v) ascorbic acid for 2 h at 37°C. After adding 10 μl of 2 mM gallic acid as an internal standard, the mixture was defatted with 1 ml hexane. Flavan-3-ols were extracted with 2 ml of ethyl acetate 4 times and dried up *in vacuo*.

High performance liquid chromatography (HiHPLC) was performed using a Hitachi D-7000 system (Hitachi, Tokyo, Japan) consisting of D-7000 command control interface, L-7455 diode array detector, L-7300 column oven, L-7100 pump and L-7200 autosampler. Chromatographic separation was achieved on a Spelco Discovery® HS PEG (φ4.6 mm×25 cm, Sigma-Aldrich) at 40°C using 0.1% aqueous formic acid (A)–acetonitrile containing 1% formic acid (B) as the mobile phase. The elution gradient worked as follows: 0.0–2.0 min, 5% B; 2.0–30.0 min, 5.0–50.0% B; 30.0–31.0 min, 50.0% B; 31.0–32.0 min, 50.0–5.0% B. The flow rate was kept at 1.0 ml/min.

*Statistical analysis*-Data are represented as the means and standard error (SE). The statistical significance of experimental observations was determined using the Dunnett or Tukey‒Kramer multiple comparison test. The level of significance was set at *p* <0.05.

## Results

**Fig 2:**
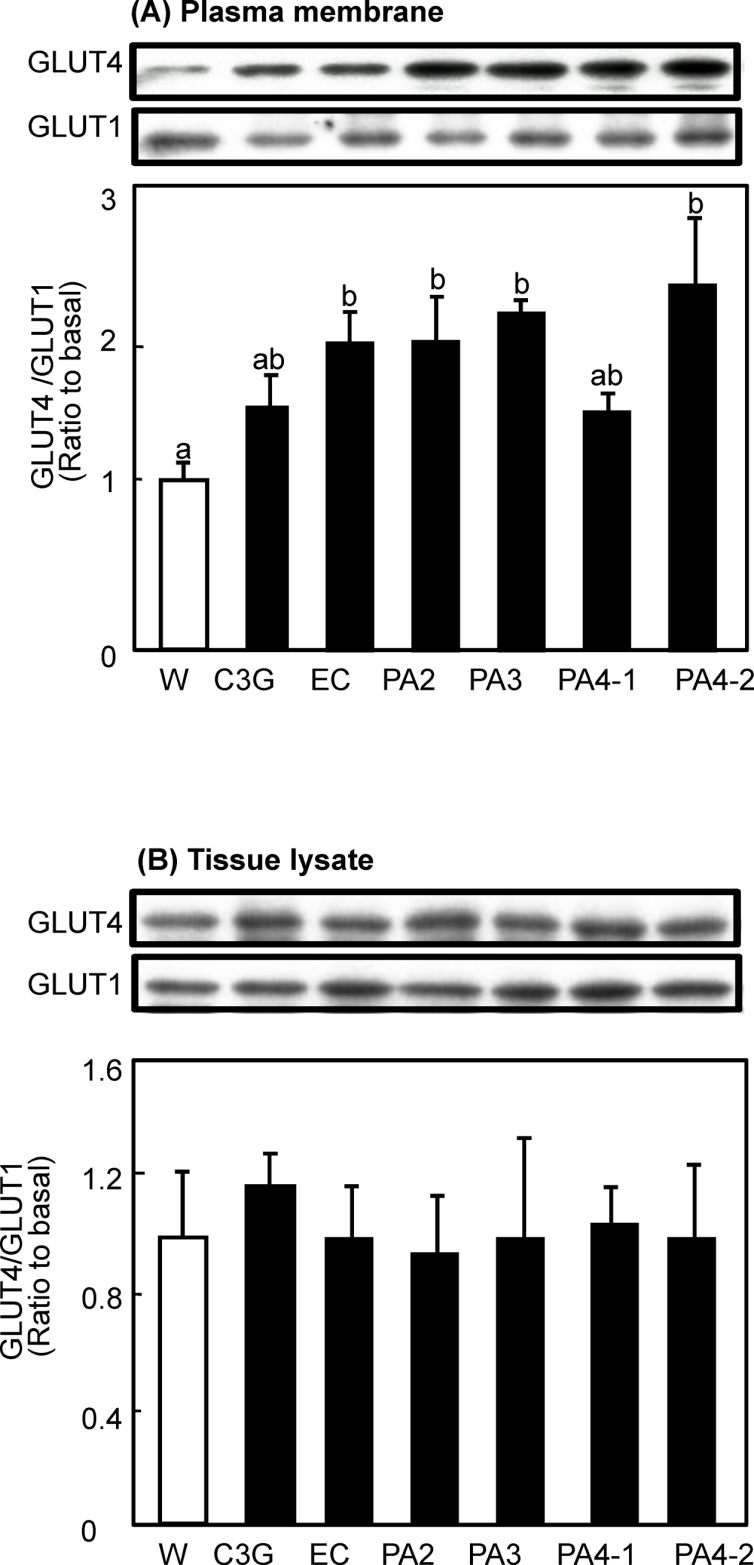
Effects of procyanidins, EC and C3G on GLUT4 translocation in skeletal muscle of mice. ICR mice were given an oral dose of procyanidins, EC, and C3G at 10 μg/kg body weight and water alone (5 mL/kg body weight) as a vehicle control. Skeletal muscle was removed 60 min after administration. The amounts of GLUT4 and GLUT1 proteins in the plasma membrane (A) and the tissue lysate (B) of the muscle were determined by immunoblotting. Each panel shows a typical result from six animals. The density of each band was analyzed and normalized to that of GLUT1. Values are means ± SE (n = 6). Different superscripted letters indicate significant differences between the groups (*p* <0.05; Tukey-Kramer multiple comparison test).

*Procyanidins promoted GLUT4 translocation to the plasma membrane in soleus muscle*-We investigated the effect of a single oral administration of procyanidins on GLUT4 translocation in the soleus muscle of ICR mice. As shown in Fig 2A, EC, PA2, PA3 and PA4-2 significantly increased GLUT4 translocation by 195%, 195%, 213% and 232%, respectively, compared with the water given controls and PA4-2 showed the strongest effect. PA4-1 and C3G also increased GLUT4 translocation by 155% and 118%, respectively, without statistical significance. On the other hand, GLUT1 in the plasma membrane was unchanged. Also, none of the compounds affected the expression level of GLUT4 in the cell lysate (Fig 2B).

**Fig 3:**
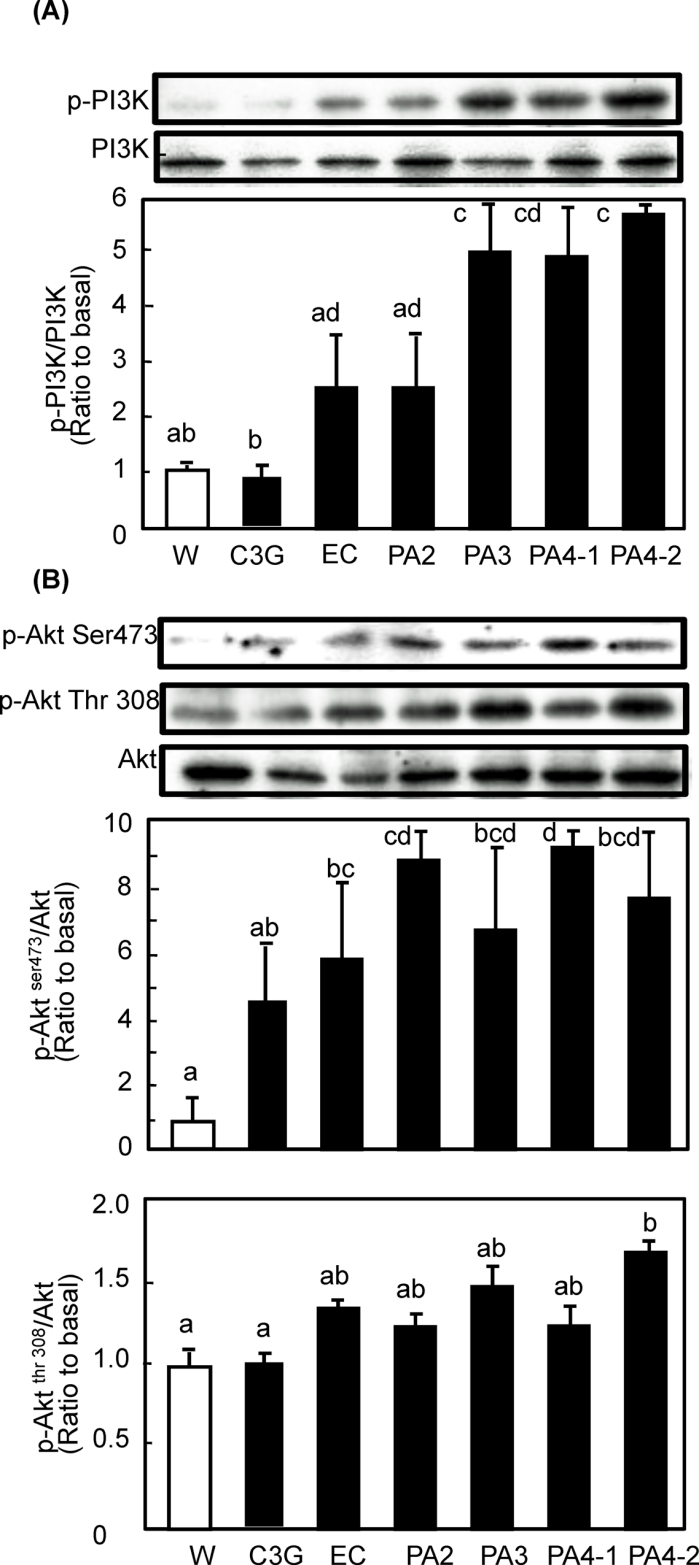
Effect of procyanidins, EC and C3G on phosphorylation of PI3K and Akt in skeletal muscle of mice. ICR mice were treated as described in Fig 2. Tissue lysate of skeletal muscle was prepared 60 min after the administration. Then, these lysate was subjected to immunoblotting analysis to determine (A) p-PI3K and PI3K; and (B) p-Akt serine 473 and threonine 308 and Akt. Each panel shows a typical result from six animals. The density of each band was analyzed and shown in the bottom panel. Values are means ± SE (n = 6). Different superscripted letters indicate significant differences between the groups (*p* <0.05; Tukey-Kramer multiple comparison test).

**Fig 4:**
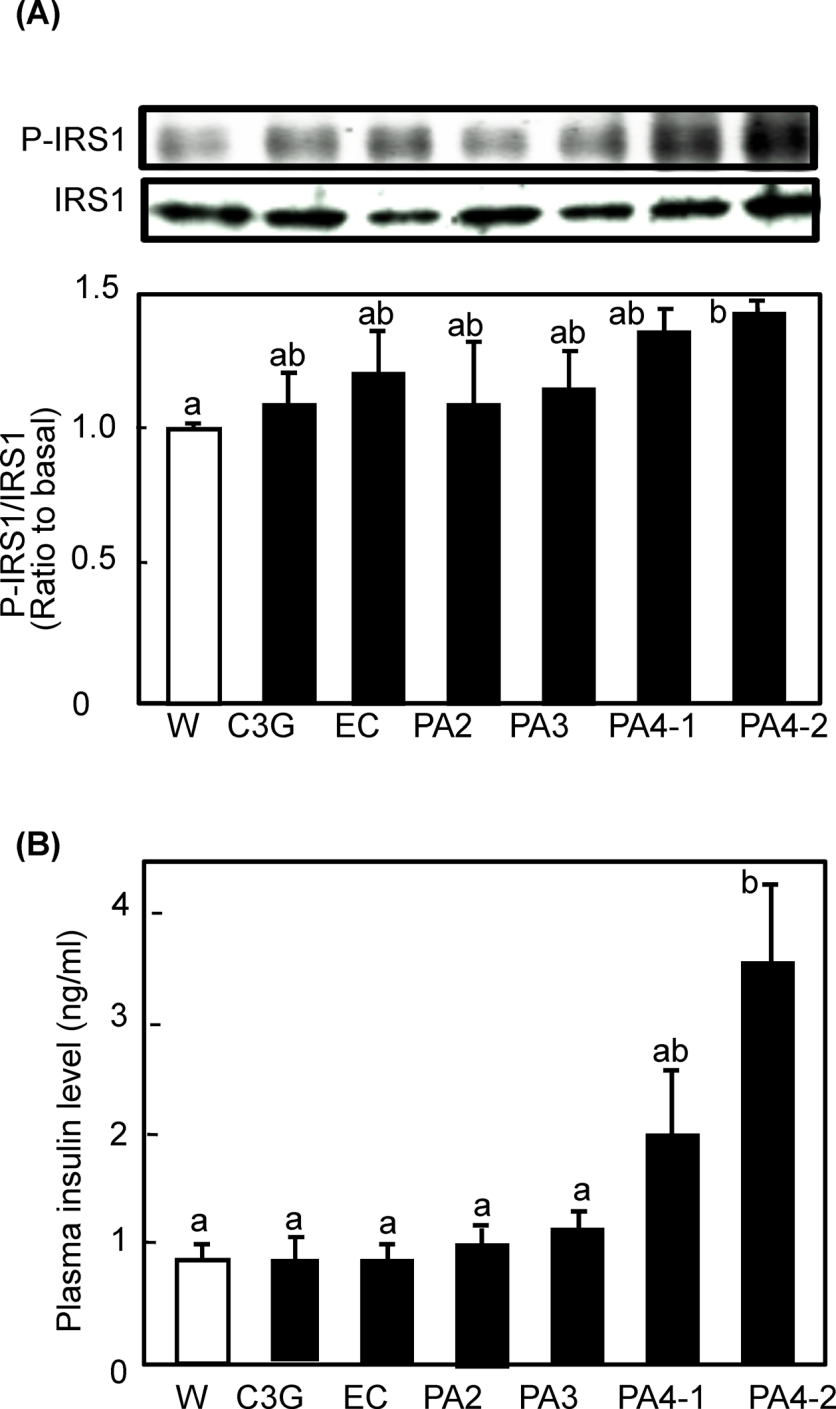
Effect of procyanidins, EC and C3G on phosphorylation of IRS-1 in skeletal muscle of mice. ICR mice were treated as described in Fig 2. (A) Tissue lysate of skeletal muscle was prepared 60 min after the administration and subjected to immunoblotting analysis to determine p-IRS-1 and IRS-1. Each panel shows a typical result from six animals. The density of each band was analyzed and shown in the bottom panel. (B) Level of plasma insulin was determined by an ELISA kit. Values are means ± SE (n = 6). Different superscripted letters indicate significant differences between the groups (*p* <0.05; Tukey-Kramer multiple comparison test).

*Procyanidin activated insulin and AMPK signaling pathways in soleus muscle of mice***-** Insulin- and/or AMPK-signaling pathways are known to be involved in GLUT4 translocation [15]. To elucidate the mechanisms by which procyanidins promote GLUT4 translocation, phosphorylation of IRS-1, PI3K, Akt, and AMPK were investigated in soleus muscle of mice after administration of each compound. Regarding PI3K/Akt-dependent signaling pathway, all procyanidins including monomer EC were found to promote phosphorylation of PI3K (Fig 3). Trimeric and tetrameric procyanidins significantly increased phosphorylation of PI3K, while others showed an increasing tendency without statistical significance. The phosphorylation level was dependent on the degree of polymerization of the compounds. In the case of Akt, one of the target molecules of PI3K, procyanidins and EC also significantly promoted the phosphorylation of Akt1 at Serine 473. However, all compounds failed phosphorylation of Akt1 at Threonine 308 except for PA 4–2, which significantly promoted phosphorylation. These results indicate that procyanidins and EC promote GLUT4 translocation by activating both the PI3K/Akt-dependent-signaling pathway. In particular, PA 4–2 showed the strongest effect on the activation of the signaling pathways with full activation of Akt1. To clarify the effect of procyanidins on upstream event, the phosphorylation level of IRS-1 and plasma insulin level were measured. As the result, only PA 4–2 significantly promoted the phosphorylation of IRS-1 and increased plasma insulin level (Fig 4). In the same animals, plasma glucose level was remained unchanged (data not shown).

**Fig 5:**
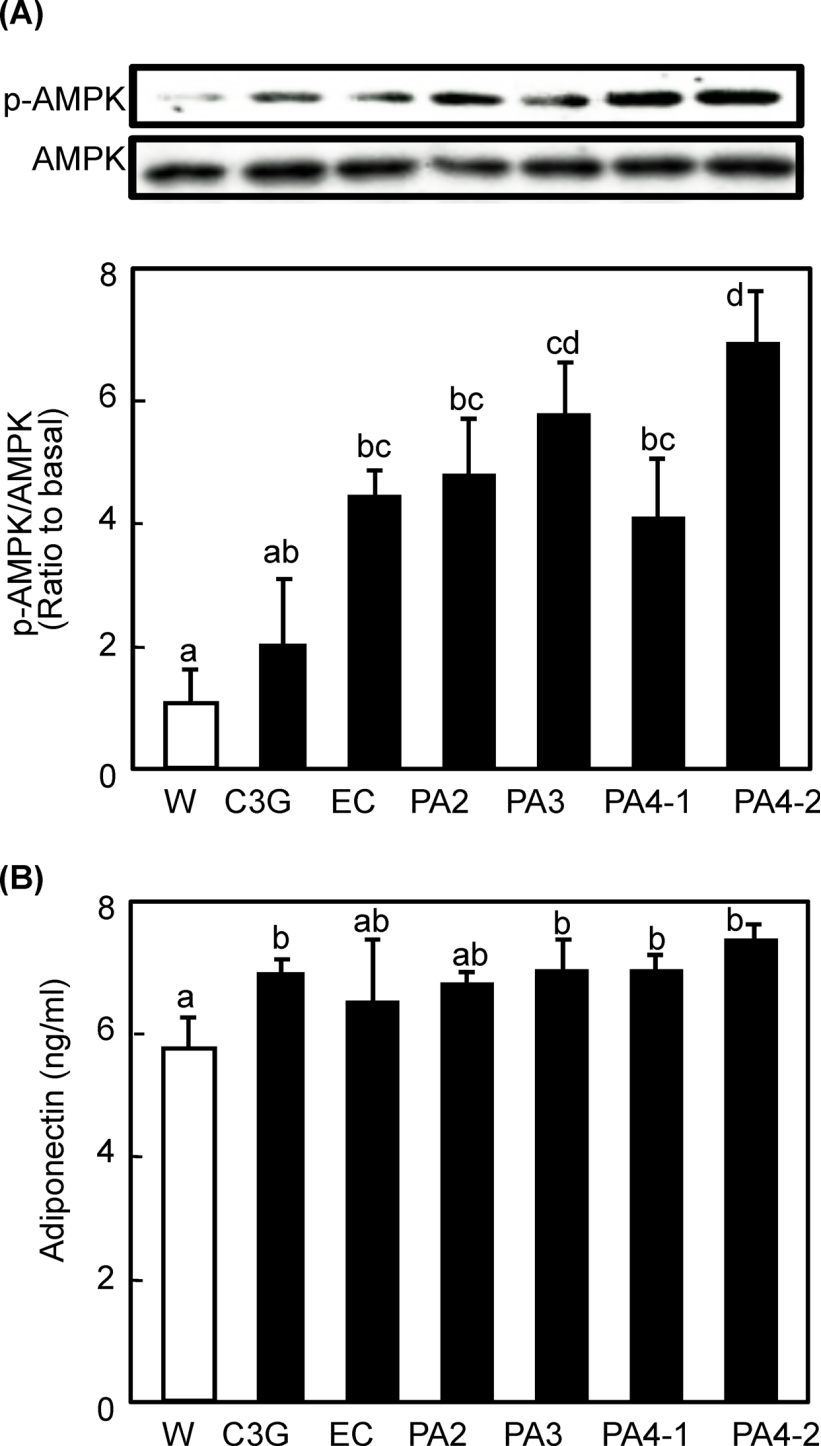
Effects of procyanidins, EC and C3G on AMPK phosphorylation in skeletal muscle of mice. ICR mice were treated as described in Fig 2. (A) Tissue lysate of skeletal muscle was prepared 60 min after the administration and subjected to immunoblotting analysis to determine p-AMPK and AMPK. Each panel shows a typical result from six animals. The density of each band was analyzed and shown in the bottom panel. (B) Level of plasma adiponectine was determined by an ELISA kit. Values are means ± SE (n = 6). Different superscripted letters indicate significant differences between the groups (*p* <0.05; Tukey-Kramer multiple comparison test).

As to AMPK, all compounds except C3G significantly promoted phosphorylation of AMPK in a polymerization-degree dependent manner (Fig 5). The expression level of AMPK did not alter with any of the treatments. Since adiponectin is a one of the candidates on upstream factor for AMPK, we measured plasma adiponectine level and found that trimeric and tetrameric procyanidins significantly increased the level, while others showed an increasing tendency without statistical significance.

**Fig 6:**
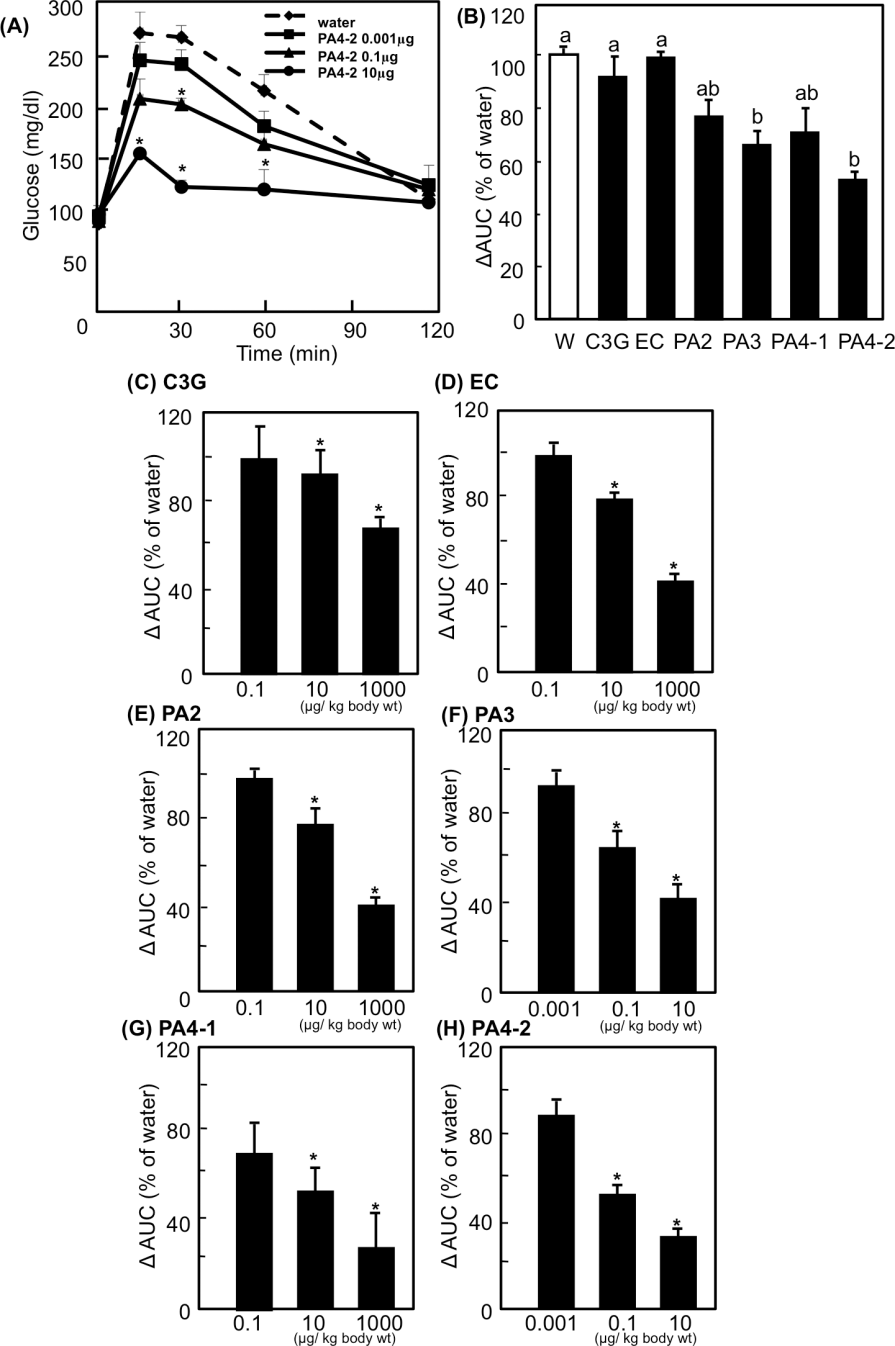
Effects of procyanidins, EC and C3G on the plasma glucose level in an oral glucose tolerance test. ICR mice were dosed orally with 0.001 to 1000 μg/kg body weight of polyphenols or water (5 mL/kg body weight). Sixty minutes after the administration, the mice were orally given glucose solution (1 g/kg body weight) followed by plasma glucose measurements at 0, 15, 30, 60 and 120 min. (A) Results of oral glucose tolerance tests were shown after the treatment with 0.001 (■), 0.1 (▲) or 10 (●) μg/kg body weight of PA 4–2 or water [5 mL/kg body weight; (●)]. Values are means ± SE (n = 6). Values of area under the curve (AUC) calculated from the treatment with 0.001 to 1000 μg/kg body weight of polyphenols are shown in the panels B to H. (B) AUC from the treatment with 0.1μg/kg body weight of polyphenols. (C-H) AUC from the treatment with each polyphenol at different concentrations. Values are means ± SE (n = 6). *Significantly different from the corresponding control group (*p* <0.05; Dunnett’s test in the panels A and C to H). Different superscripted letters indicate significant differences between the groups (*p* <0.05; Tukey-Kramer multiple comparison test in the panel B).

*Procyanidins improve postprandial hyperglycemia in OGTT*-We performed OGTT to evaluate the effect of procyanidins on postprandial hyperglycemia. One of the typical results of OGTT is shown in Fig 6A. Plasma glucose level in the control group, which was given glucose alone, increased in response to oral glucose loading and reached maximum after 15 min following the intake of glucose. The glucose level then decreased with time and recovered to the normal level by 120 min. Pre-administration of PA 4–2 suppressed postprandial hyperglycemia in a dose-dependent manner. When mice received 10 μg/kg body weight of PA 4–2, a significant effect was observed at 15, 30 and 60 min after the glucose loading. PA 4–2 at 0.1 μg/kg body weight also showed a significant reduction of the glucose level 30 min after the glucose loading.

The suppression effect of procyanidins, EC and C3G was estimated after calculation of the area under the curve (AUC) of the plasma glucose levels. Fig 6B showed comparison of the suppression effect between compounds; 0.1 μg/kg body weight of procyanidins reduced AUC, particularly PA3 and PA4-2 significantly reduced AUC compared with the water group. All of the compounds significantly suppressed the postprandial increased plasma glucose levels at 10 μg and 1000 μg/ kg body weight in a dose-dependent manner (Fig 6C–6H).

*Effects of procyanidins on intestinal α-glucosidase activity*-Oligomeric procyanidins from grape-seed were reported to inhibit the enzymatic activity of intestinal α-glucosidases, including maltase and sucrase *in vitro* [28]. It is possible that a similar effect may be responsible for the decrease in postprandial blood glucose level. Therefore, we measured the inhibitory effects of procyanidins, EC and C3G on α-glucosidase activity *in vivo*. All of the compounds did not affect α-glucosidase activity (data not shown).

**Fig 7:**
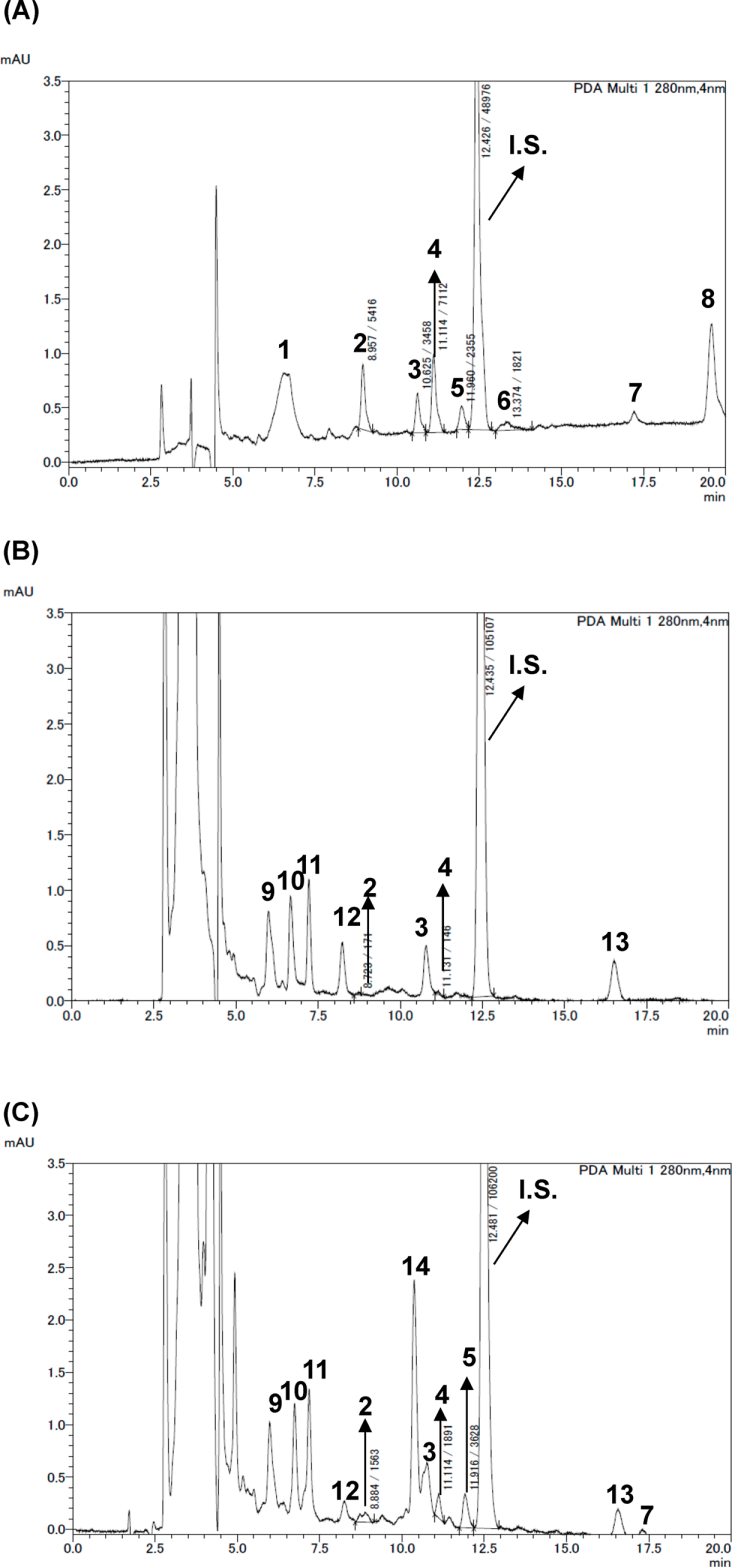
Concentrations of flavan-3-ols in plasma of mice given black soybean seed coat extract (BE). ICR mice were given an oral dose of BE in water at 1 g/kg body weight. The mice were sacrificed 60 min after the dose of BE and blood collected from a cardiac puncture. And then, these plasma samples were used for measurement of flavan-3ols by HPLC. (A) a typical HPLC chromatogram of BE (B) that of flavan-3-ols extracted from BE-dosed mice plasma, and (C) that of flavan-3-ols extracted from BE-dosed mice plasma after treatment with β-glucronidase. 1.C3G. 2.PA2. 3.Unknown. 4. EC. 5.PA3. 6.PA4-2. 7.Unknown. 8.Unkown 9.Unknown. 10.Unknown. 11. Unknown. 12.Unknown. 13.Unknown. 14.Unknown. IS. Internal standard.

*Plasma concentration of procyanidins after administration of BE and its polyphnols* -To investigate whether procyanidins and EC are incorporated into the body, the plasma of mice given BE at 1 g/kg body weight were applied to HPLC (Fig 7). Although dose of BE used in this experiment was at least 1000-fold higher than that in OGTT and immunoblotting experiments, PA4-2 was not detected in the plasma. Aglycone and its conjugated form of EC were detected at 1.94 ± 0.61 and 4.55 ± 2.03 μM, respectively; those of PA2 were 3.22 ± 1.46 and 2.11 ± 1.25 μM, respectively; and those of PA3 were 0.15 ± 0.19 and 1.63 ± 0.69 μM, respectively, in the plasma. Moreover, we measured plasma concentration of flavan-3-ols in mice after given each compound at 10 μg/kg body weight. After 1 h-administration, aglycone and its conjugated form of EC were detected at 2.81 ± 0.71 and 5.80 ± 0.93 nM, respectively, whereas neither aglycone nor conjugated form were detected after any administration of procyanidin.

## Discussion

Chronic hyperglycemia has become a serious health problem in many countries, which is thought to be due to over-eating and physical inactivity. Hyperglycemia is a risk factor for the onset of cardiovascular disease. The bioactive compounds in food including polyphenols contribute to anti-hyperglycemic effects [29–32]. In the present study, we investigated whether purified procyanidin compounds prevent hyperglycemia in ICR mice. We found that procyanidins, in particular tetramer cinnamtannin A2 (PA 4–2) prevented postprandial hyperglycemia at least in part by stimulating GLUT4 translocation to the plasma membrane of skeletal muscle (Figs 2 and 6). Black soybeans contain 0.2% BE and BE contains 6.11% EC, 6.06% procyanidin B2 (PA2), 3.94% procyanidin C1 (PA3) and 1.18% cinnamtannin A2 (PA 4–2). From these data and the effective dose in mice (10 μg/kg body weight: Fig 6C), effective dose range of black soybeans is from 5.7 (for EC) to 29.7 g beans [for cinnamtannin A2 (PA 4–2)]/day/70 kg body weight in human. Moreover, back soybeans contain a mixture of these compounds. We speculate 10 g/day/person of black soybeans is suitable amount for human study in future. Thus, procyanidins may prevent postprandial hyperglycemia at a physiological concentration range.

Trimeric and tetrameric procyanidins significantly activated both the PI3K/Akt- and AMPK-dependent signaling pathways to induce GLUT4 translocation (Figs 3–5). Concurrently, we also found that trimeric and tetrameric procyanidins significantly increases plasma adiponectin level (Fig 5B). Adiponectin, is known to activate AMPK through the activation of LKB1 upon binding to its receptor [33]. Moreover, adiponectin plays a protective role in insulin resistance [34] and adiponectin induces insulin secretion [35]. These results suggest that trimeric and tetrameric procyanidins activate AMPK signaling pathway and improve insulin sensitivity through adiponectine secretion. Further study is needed to clearly the mechanism by which procyanidins activate AMPK. In this study, cinnamtannin A2 (PA4-2) showed the strongest activity and underlying molecular mechanism of this compound is different from others, because only cinnamtannin A2 (PA4-2) promoted the secretion of insulin and increased phosphorylation of IRS-1 and Akt1 Threonine 308 in addition to Serine 473. These results strongly suggest that cinnamtannin A2 possesses insulin mimetic activity. To our knowledge, this is the first report to show that purified procyanidin compound suppress postprandial hyperglycemia through promoting GLUT4 translocation by both insulin- and AMPK-dependent signaling pathways in skeletal muscle of mice. Our findings provide evidence that cinnamtannin A2 (PA4-2), is effective food component for the prevention of diabetes mellitus.

GLUT4 translocation in muscle cells is a pivotal role in maintenance of glucose homeostasis, in particular lowering the blood glucose level in postprandial state. It is known that two main signaling pathways are involved in GLUT4 translocation in skeletal muscle, the insulin- and AMPK-dependent pathways [11, 12]. In the insulin-dependent pathway, the binding of insulin to its α-subunits induces the autophosphorylation of the β-subunits [11, 36], leading to the sequential activation of a number of docking proteins, including insulin receptor substrates [36], PI3K, Akt [37] and aPKC [38]. Signals then transmit to intracellular vesicles containing GLUT4, and this leads to the translocation, docking and fusion of GLUT4 on the plasma membrane [11, 36]. Although the insulin-dependent pathway mainly contributes to lowering the blood glucose level in postprandial state, the AMPK-dependent pathway also plays an important role in GLUT4 translocation as the insulin-independent pathway [13, 14]. AMPK is an energy sensor that regulates both lipid and carbohydrate homeostasis, and impairments in its function have been linked with the progression of metabolic disorders [37]. AMPK is activated both in response to exercise and muscle contraction through an increase in AMP concentration [38]. AMP activates AMPK by binding to the two CBS domains on the γ-subunit, which activates AMPK directly by an allosteric mechanism and indirectly activating phosphorylation on Thr172 on the α-subunit by upstream kinase(s), including LKB1, resulting in promotion of GLUT4 translocation [37–39].

In this study, we found that procyanidins promoted GLUT4 translocation through both the PI3K/Akt- and AMPK-dependent signaling pathways in skeletal muscle of mice *in vivo* (Figs 2–5). On the contrary, results from our previous study [21] have demonstrated that procyanidins promote GLUT4 translocation through AMPK-dependent signaling pathway without affecting phosphorylation of Akt in L6 myotubes. Curcumin [40] and anthocyanin [29] also promoted GLUT4 translocation in muscle cells through an AMPK-dependent pathway. We have previously reported that EGCg promotes GLUT4 translocation in L6 cells through the PI3K-dependent pathway [24], whereas other studies have reported that EGCg promotes through the AMPK-dependent signaling pathway [22, 23]. Resveratrol [19] and its metabolite, piceatannol [41], stimulate GLUT4 translocation in L6 myotubes by activating the AMPK-dependent signaling pathway. Resveratrol has also been reported to activate the Akt pathway in patients with type 2 diabetes [42]. Moreover, kaempferide [43] promotes GLUT4 translocation in L6 myotubes by activating both PI3K- and AMPK-dependent dual-signaling pathways. On the other hand, prenylated chalcones, 4-hydroxyderricin and xanthoangelol [44] and cardamonin [45] were found to stimulate GLUT4 translocation, but not through either the PI3K/Akt or AMPK pathways. Therefore, signaling pathways for GLUT4 activated by polyphenol are complex, and even the same compound shows different mechanisms in different experimental conditions.

In this study, the anti-postprandial hyperglycemic effects, as estimated by OGTT, were the greatest with cinnamtannin A2 (PA4-2) of all the compounds studied (Fig 6), and that GLUT4 translocation (Fig 2) occurred through activation of both the insulin- and AMPK-signaling pathways in skeletal muscle (Figs 3–5). However, our previous report [6] demonstrated that long-term (13 weeks) intake of procyanidin-rich cacao polyphenols suppressed high-fat diet induced hyperglycemia through the promotion of GLUT4 translocation in skeletal muscle, accompanied by PI3K-indepdendent and AMPK-depdendent pathway. This discrepancy suggests that procyanidins are primarily responsible for the beneficial actions, partially through the modulation of the enteroendocrine system *in vivo*, such as the incretin effect. Results in the present study have demonstrated that cinnamtannin A2 (PA4-2) specifically increases insulin secretion in the plasma and phosphorylation of IRS1 in muscle. These results are coincide with those in our previous study [46], demonstrating that only cinnamtannin A2 (PA4-2) among procyanidins increases glucagon-like peptide-1 (GLP-1) and insulin secretion in the plasma, resulting in the phosphorylation of IRβ and IRS1 in muscle after 60 min p.o.-administration. Although we did not measure secretion of GLP-1 in this study, we assume that GLP-1 may increase in plasma after administration of cinnamtannin A2 (PA4-2). With respect to the incretin effect of procyanidins, grape seed-derived procyanidins decrease dipeptidyl-peptidase 4, an inhibitor of GLP-1 degradation, activity and expression [32, 47]. Resveratrol has also been reported to increase glucose-induced GLP-1 secretion in high-fat fed mice with diabetes [48]. Together, results in this study and these previous ones suggest that polyphenols including procyanidins may have the potential to show the incretin effect. Moreover, cinnamtannin A2 (PA4-2) was not detected in the plasma after administration of huge amounts of procyanidin-rich black soybean polyphenols, though certain amounts of aglycone and conjugation forms of EC, procyanidin B2 and procyanidin C1 were detected in the plasma (Fig 7). This results support previously reported ones, that monomer, dimers and trimers are absorbed into the body [49], unlike the tetramers, which are thought to have little or no absorption into the body. Further study is needed to clarify how cinnamtannin A2 (PA4-2) promotes secretion of GLP-1 from intestinal cells.

Inhibition of α-glucosidase activity is an important mechanism that suppresses postprandial hyperglycemia [50]. However, our results demonstrated that procyanidin did not inhibit intestinal α-glucosidase activity *in vivo*. In our previous report, we showed that long-term intake of green and black tea [51] and cacao liquor procyanidins [6] suppressed hyperglycemia by modulating the expression and translocation of GLUT4 without inhibiting α-glucosidase activity, similar to that found in the current study. Many *in vitro* studies have shown that certain polyphenols, including anthocyanins, catechins, theaflavins, quercetin and luteolin, can inhibit intestinal α-glucosidase activity [52–56]. As regards procyanidins, it was reported that the inhibitory effects of oligomeric procyanidins on α-glucosidase activity are dependent on their molecular weight, since the tetrameric and hexameric procyanidins are more potent inhibitors than the dimeric and trimeric procyanidins *in vitro* [57]. There is a discrepancy between the *in vivo* and *in vitro* results of the inhibitory effect of polyphenols on α-glucosidase activity, although it was reported that rutin inhibited α-glucosidase activity in both *in vivo* and *in vitro* [52].

In conclusion, our current findings provide strong evidence that procyanidin cinnamtannin A2 (PA4-2) prevent hyperglycemia and ameliorate glucose tolerance through promoting GLUT4 translocation and enhancing glucose uptake by incretin hormone GLP-1 driven activation of insulin signaling pathway. Moreover, procyanidins including cinnamtannin A2 (PA4-2) have ability to promote GLUT4 translocation by activating both insulin- and AMPK-dependent dual-signaling pathways, independent of incretin effect. Therefore, procyanidins are a promising food component for the prevention of hyperglycemia and diabetes mellitus.

## Abbreviations

AMPK: AMP-activated protein kinase
AUC: area under the curve
BE: black soybean seed coat extract
C3G: Cyanidin-3-O-β-glucoside
EC: (−)-epicatechin
EGCg: (−)-epigallocatechin-3-gallate
GLUT4: glucose transporter 4
IR: insulin receptor
IRS: insulin receptor substrate
OGTT: oral glucose tolerance test
PI3K: phosphoinositide 3-kinase
PA2: procyanidin B2
PA3: procyanidin C1
PA4-1: EC-(4β–6)-EC-(4β–8)-EC-(4β–8)-EC
PA4-2: cinnamtannin A2
